# {*N*,*N*′-Bis[1-(2-pyrid­yl)ethyl­idene]ethane-1,2-diamine-κ^4^ *N*,*N*′,*N*′′,*N*′′′}bis­(trifluoro­methane­sulfanato-κ*O*)copper(II)

**DOI:** 10.1107/S1600536808033151

**Published:** 2008-10-18

**Authors:** Simon J. Coles, Abdurrahman Sengul, Ozgur Kurt, Safinaz Altin

**Affiliations:** a School of Chemistry, University of Southampton, Southampton SO17 1BJ, England; b Department of Chemistry, Faculty of Arts and Sciences, Zonguldak Karaelmas University, 67100 Zonguldak, Turkey

## Abstract

A discrete neutral Cu^II^ complex, [Cu(CF_3_SO_3_)_2_(C_16_H_18_N_4_)], has been derived from the symmetrical tetra­dentate Schiff base, *N*,*N*′-bis­[1-(pyridin-2-yl)ethyl­idene]ethane-1,2-diamine. The copper centre assumes a tetra­gonally distorted pseudo-octa­hedral geometry with the O atoms of two trifluoro­methane­sulfonate anions coordinated weakly in the axial positions. The Cu—N distances lie in the range 1.941 (3)–2.011 (3) Å and the Cu—O distances are 2.474 (3) and 2.564 (3) Å.

## Related literature

For general background, see: Gourbatsis *et al.* (1999 ▶); Hamblin *et al.* (2002 ▶); Menteş *et al.* (2007 ▶); Szklarzewicz & Samotus (2002 ▶). For related synthesis, see:  Hanack *et al.* (1988 ▶); Luo *et al.* (1993 ▶); Marks (1990 ▶). For related structural characteristics, see: Bowyer *et al.* (1998 ▶); Gourbatsis *et al.* (1998 ▶); Cremer & Pople (1975 ▶); Fielden *et al.* (2006 ▶); Haynes *et al.* (1988 ▶); Şengül & Büyükgüngör (2005 ▶).
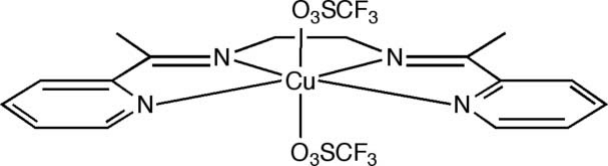


## Experimental

### 

#### Crystal data

[Cu(CF_3_SO_3_)_2_(C_16_H_18_N_4_)]
*M* *_r_* = 628.02
Monoclinic, 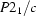

*a* = 9.2228 (4) Å
*b* = 25.5574 (13) Å
*c* = 9.8189 (5) Å
β = 94.961 (3)°
*V* = 2305.75 (19) Å^3^
*Z* = 4
Mo *K*α radiation
μ = 1.22 mm^−1^
*T* = 120 (2) K
0.35 × 0.2 × 0.06 mm

#### Data collection

Bruker Nonius KappaCCD area-detector diffractometer
Absorption correction: multi-scan (*SADABS*; Sheldrick, 2007 ▶) *T*_min_ = 0.78, *T*_max_ = 0.93
18769 measured reflections
5052 independent reflections
4062 reflections with *I* > 2σ(*I*)
*R*_int_ = 0.043

#### Refinement

*R*[*F* ^2^ > 2σ(*F* ^2^)] = 0.046
*wR*(*F* ^2^) = 0.118
*S* = 1.09
5052 reflections
334 parameters
H-atom parameters constrained
Δρ_max_ = 0.43 e Å^−3^
Δρ_min_ = −0.64 e Å^−3^

### 

Data collection: *DENZO* (Otwinowski & Minor, 1997 ▶); cell refinement: *DENZO* and *COLLECT* (Nonius, 1998 ▶); data reduction: *DENZO* and *COLLECT*; program(s) used to solve structure: *SHELXS97* (Sheldrick, 2008 ▶); program(s) used to refine structure: *SHELXL97* (Sheldrick, 2008 ▶); molecular graphics: *PLATON* (Spek, 2003 ▶); software used to prepare material for publication: *publCIF* (Westrip, 2008 ▶).

## Supplementary Material

Crystal structure: contains datablocks global, I. DOI: 10.1107/S1600536808033151/bg2203sup1.cif

Structure factors: contains datablocks I. DOI: 10.1107/S1600536808033151/bg2203Isup2.hkl

Additional supplementary materials:  crystallographic information; 3D view; checkCIF report

## Figures and Tables

**Fig. 1.**
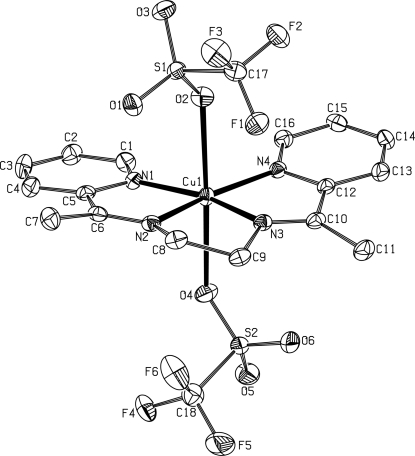
Molecular structure of the title compound (50% probability displacement ellipsoids).

## supplementary crystallographic information

### Comment

Recently, the coordination chemistry of di-Schiff bases derived from 2-pyridyl ketones or aldehydes has generated a great deal of interest (Hamblin *et al.*, 2002; Gourbatsis *et al.*, 1998; Szklarzewicz & Samotus, 2002; Mentes *et al.*, 2007). These studies have been mostly stimulated by an interest in modelling the enzyme, copper-zinc superoxide dismutase (SOD) (Luo *et al.*, 1993) and also for the synthesis of metal containing polymers with interesting optical, magnetic and electrical properties (Hanack *et al.*, 1988; Marks, 1990). It has also been found that such tetradentate Schiff base ligands may form complexes with different nuclearity according to the coordination preferences of the metal centre (Fielden *et al.*, 2006).

Our interest in the ligand, *L*, (Scheme 2) was stimulated by the analogy between its donor set and that of the pyridylmethylketazine (*L*^1^) and 2-pyridinealdazine (*L*^2^) system which form triple-stranded helical complexes with the formula [*M*_2_(*L*)_3_]^4+^ (*M* = Co, Fe and Ni). The helical complexes were shown to undergo exchange reactions on standing to form mono-nuclear complexes [*M*(*L*)_2_]^2+^ in which the ligand twists to coordinate as tridentate with non-coordinated imine residue (Hamblin *et al.*, 2002). Mononuclear species are favoured by coordination to octahedral metal centres whose equatorial sites are occupied by N4 donor set of the bis(axial) ligand, and their axial sites being occuppied by solvent molecules or counterions. In addition, dinuclear metal complexes are favoured by the four-coordinate tetrahedral metal centres whose *ca* 90° twist angle provides good geometric match for the bis(equatorial) ligand (Fielden *et al.*, 2006).

Recently, the single-crystal X-ray analysis of *L* was reported (Mentes *et al.*, 2007). The molecule adopts a centrosymmetric *trans* geometry and forms a dinuclear complex by reacting with Mo(CO)_6_. The reaction of *L* with Zn*X*_2_ (*X* = Cl or Br) in tetrahydrofuran yielded an octahedral complex [Zn*X*_2_(*L*)] (Gourbatsis *et al.*, 1999), whereas by reacting with a silver(I) cation the double-stranded helical complex [Ag_2_(*L*)_2_][BF_4_]_2_ (Bowyer *et al.*, 1998) is formed. The synthesis of copper(II) complex by using Cu(NO_3_)_2_.3H_2_O resulted in the tetragonally distorted octahedral complex, [Cu(*L*)(ONO_2_)(OH_2_)][NO_3_] (Gourbatsis *et al.*, 1998).

Herein we present the synthesis and structure of the complex [Cu(*L*)(OTf)_2_], (where OTf = trifluoromethanesulfonate) with a molecular structure as illustrated in Scheme 1 and Figure 1. The crystal structure is composed of discrete neutral [Cu(*L*)(OTf)_2_] units. The copper ion exhibits an elongated tetragonal octahedral CuN_4_O_2_ cromophore with four nitrogen atoms from the ligand occupying the equatorial plane and two axial oxygen atoms from the *trans*-coordinated unidentate trifluoromethanesulfonate anions. The four equatorial Cu–N distances [Cu1–N1 2.008 (3) Å, Cu1–N4 2.011 (3) Å, Cu1–N2 1.950 (2) Å, and Cu1–N3 1.944 (2) Å] are normal for this class of compounds and also very similar to those of Cu1–Npyridine 2.002 (4) and 2.022 (4) Å, and Cu1–Nimine 1.943 (4) and 1.938 (4) Å as found in [Cu(*L*)(ONO_2_)(OH_2_)][NO_3_] (Gourbatsis *et al.*, 1998). The bond lengths to the imine N atoms are slightly shorter than those to the pyridine N atoms (Table 1), which is presumably a consequence of the more effective σ donation or π back donation (Hamblin *et al.*, 2002). The coordination is of the 4 + 2 type and thus belongs to the myriad of examples of such Jahn-Teller elongated pseudo-octahedral structures (Şengül & Büyükgüngör, 2005).

The structure contains unidentate trifluoromethanesulfonate anions which are semi-coordinated to the copper ion [Cu1–O2 2.568 (3) Å and Cu1–O4 2.476 (4) Å] in the axial positions with the angle of O2–Cu1–O4 177.9 (4)°. The bonding parameters for the trifluoromethanesulfonate anions are similar to those found for [Cu(pyridine)_4_(CF_3_SO_3_)_2_] (Haynes *et al.*, 1988). For example, the trifluoromethanesulfonate anions adopt a staggered-ethane configuration about the S–C bond and the O–S–O angles [O3–S1–O1 116.01 (15)°, O3–S1–O2 113.97 (14)°, O1–S1–O2 114.93 (15)°] are greater than the C–S–O angles [C17–S1–O3 103.25 (15)°, C17–S1–O1 103.00 (15)°, and C17–S1–O2 103.25 (15)°]. The S–O bond lengths are also very similar to those found in [Cu(pyridine)_4_(CF_3_SO_3_)_2_], the S1–O2 1.450 (2) and S2–O4 1.446 (2) Å bonds involving the O atoms bound to copper being longer than those involving the terminally bound oxygen atoms [S1–O1 1.442 (2), S1–O3 1.440 (2) Å and S2–O5 1.441 (2), S2–O6 1.440 (2) Å].

The bite angles around the copper ion [N2–Cu1–N3 83.2 (2), N1–Cu1–N2 81.9 (7), N3–Cu1–N4 81.6 (7)°] are very similar to those found in [Cu(*L*)(ONO_2_)(OH_2_)][NO_3_] with the corresponding angles of 83.2 (2), 80.6 (2) and 81.4 (2)°, respectively.

In the free ligand the pyridylimine units adopt a *transoid* configuration to minimize unfavourable electronic interactions between the lone pairs of pyridine nitrogen and imine nitrogen atoms. However, in the presence of a metal ion, the pyridine rings rotate by 180° with respect to the Aryl–C bond, positioning the two nitrogen atoms of each pyridylimine moiety on the same side of the ligand. Otherwise the geometric parameters in the free ligand are very similar to those of the coordinated moiety.

The pyridylimine units are not ideally planar due to a combined effect of the ring to the metal centre and a twist induced by the ethylene bridge [Cu1, N1, N2, C1>C6 and Cu1, N3, N4, C10, C12>C16 have devaitions from the mean plane of 0.088 (6) Å and 0.106 (6) Å for N2 and N3 respectively]. From puckering analysis (Cremer & Pople, 1975) the ring formed by the metal centre, the imine N atoms and the ethylene bridge has a Q value of 0.283 (3) Å and forms a twisted envelope conformation about the C8—C9 bond. This effect has the result of pushing the methyl groups out of the ring unit plane, with C7 deviating by 0.149 (5) Å and C11 by 0.167 (6) Å and accordingly the pyridylimine units are not coplanar, with the angle formed between these planes being 12.98 (9)°.

The crystal structure does not exhibit any classical hydrogen bonds and is primarily comprised of stacked undulating sheets formed by close packing and C—H···O interactions between the SO_3_ group and pyridylimine ring H atoms.

### Experimental

The ligand, *N*,*N*'-bis-(1-pyridin-2-yl-ethylidene)-ethane-1,2-diamine (*L*) (0.213 g; 0.8 mmol) and Cu(CF3SO3)2 (0.297 g; 0.8 mmol) were dissolved in a minimum amount of methanol. The solution was stirred at room temperature for half an hour and filtered. The navy blue solution was poured into sample tubes and left for crystallization to yield very dark navy blue block crystals suitable for X-ray diffraction analysis. Anal. Calc.: C, 34.42; H, 2.89; N, 8.92. Found: C, 34.64; H, 3.07; N, 9.06%. ESI-MS (m/*z*) = 478.0 [Cu(*L*)(OTf)]^+^.

### Refinement

All non H atoms were refined anisotropically. All hydrogen atoms were fixed in idealized positions [0.98 Å (CH_3_), 0.99 Å (CH_2_) & 0.95 Å (CH)] and refined using the riding model with *U*_iso_(H) set to 1.2 or 1.5*U*_eq_(carrier) for CH or CH_2_ and CH_3_ respectively. When including H atoms, methyl groups were allowed to rotate to enable matching with electron density maxima.

### Crystal data

**Table:**

[Cu(CF_3_SO_3_)_2_(C_16_H_18_N_4_)]	*F*(000) = 1268
*M**_r_* = 628.02	*D*_x_ = 1.809 Mg m^−^^3^
Monoclinic, *P*2_1_/*c*	Mo *K*α radiation, λ = 0.71073 Å
Hall symbol: -P 2ybc	Cell parameters from 26348 reflections
*a* = 9.2228 (4) Å	θ = 2.9–27.5°
*b* = 25.5574 (13) Å	µ = 1.22 mm^−^^1^
*c* = 9.8189 (5) Å	*T* = 120 K
β = 94.961 (3)°	Block, blue
*V* = 2305.75 (19) Å^3^	0.35 × 0.2 × 0.06 mm
*Z* = 4	

### Data collection

**Table:**

Bruker–Nonius KappaCCD area-detector diffractometer	4062 reflections with *I* > 2σ(*I*)
φ and ω scans	*R*_int_ = 0.043
Absorption correction: multi-scan (*SADABS*; Sheldrick, 2007)	θ_max_ = 27.5°, θ_min_ = 3.0°
*T*_min_ = 0.78, *T*_max_ = 0.93	*h* = −11→11
18769 measured reflections	*k* = −30→33
5052 independent reflections	*l* = −12→12

### Refinement

**Table:**

Refinement on *F*^2^	0 restraints
Least-squares matrix: full	H-atom parameters constrained
*R*[*F*^2^ > 2σ(*F*^2^)] = 0.046	*w* = 1/[σ^2^(*F*_o_^2^) + (0.0383*P*)^2^ + 4.934*P*] where *P* = (*F*_o_^2^ + 2*F*_c_^2^)/3
*wR*(*F*^2^) = 0.118	(Δ/σ)_max_ = 0.001
*S* = 1.09	Δρ_max_ = 0.43 e Å^−^^3^
5052 reflections	Δρ_min_ = −0.64 e Å^−^^3^
334 parameters	

### Special details

**Table:**

Geometry. All e.s.d.'s (except the e.s.d. in the dihedral angle between two l.s. planes) are estimated using the full covariance matrix. The cell e.s.d.'s are taken into account individually in the estimation of e.s.d.'s in distances, angles and torsion angles; correlations between e.s.d.'s in cell parameters are only used when they are defined by crystal symmetry. An approximate (isotropic) treatment of cell e.s.d.'s is used for estimating e.s.d.'s involving l.s. planes.

### Fractional atomic coordinates and isotropic or equivalent isotropic displacement parameters (Å^2^)

**Table:**

	*x*	*y*	*z*	*U*_iso_*/*U*_eq_	
Cu1	0.36217 (4)	0.096617 (15)	0.13089 (4)	0.01732 (17)	
S1	0.61344 (9)	0.13260 (3)	−0.13780 (9)	0.0201 (2)	
S2	0.11834 (9)	0.11566 (4)	0.40390 (9)	0.0216 (2)	
F1	0.6938 (2)	0.20219 (8)	0.0510 (2)	0.0335 (5)	
F2	0.8673 (2)	0.15164 (8)	−0.0042 (2)	0.0339 (6)	
F3	0.7879 (2)	0.21298 (8)	−0.1408 (3)	0.0395 (6)	
F4	−0.1392 (2)	0.14451 (8)	0.2941 (2)	0.0329 (5)	
F5	−0.0500 (3)	0.19350 (10)	0.4589 (3)	0.0529 (8)	
F6	0.0325 (2)	0.19887 (9)	0.2612 (3)	0.0451 (7)	
O1	0.5026 (3)	0.16759 (10)	−0.1968 (3)	0.0294 (6)	
O2	0.5698 (3)	0.10044 (9)	−0.0264 (3)	0.0246 (6)	
O3	0.6956 (3)	0.10514 (10)	−0.2337 (3)	0.0293 (6)	
O4	0.1560 (3)	0.09377 (9)	0.2761 (3)	0.0260 (6)	
O5	0.0389 (3)	0.08055 (11)	0.4852 (3)	0.0321 (6)	
O6	0.2334 (3)	0.14490 (11)	0.4778 (3)	0.0349 (7)	
N1	0.2386 (3)	0.05333 (10)	−0.0051 (3)	0.0169 (6)	
N2	0.2689 (3)	0.15379 (10)	0.0252 (3)	0.0188 (6)	
N3	0.4497 (3)	0.15332 (10)	0.2410 (3)	0.0177 (6)	
N4	0.4951 (3)	0.05365 (10)	0.2608 (3)	0.0164 (6)	
C1	0.2187 (4)	0.00137 (13)	−0.0122 (4)	0.0214 (7)	
H1	0.2688	−0.0198	0.0531	0.026*	
C2	0.1266 (4)	−0.02194 (14)	−0.1130 (4)	0.0252 (8)	
H2	0.1149	−0.0581	−0.1155	0.030*	
C3	0.0525 (4)	0.00934 (14)	−0.2097 (4)	0.0278 (8)	
H3	−0.0091	−0.0055	−0.2791	0.033*	
C4	0.0702 (4)	0.06320 (14)	−0.2027 (4)	0.0251 (8)	
H4	0.0187	0.0848	−0.2659	0.030*	
C5	0.1647 (4)	0.08421 (14)	−0.1013 (4)	0.0184 (7)	
C6	0.1917 (4)	0.14160 (13)	−0.0857 (4)	0.0192 (7)	
C7	0.1385 (4)	0.17828 (14)	−0.1956 (4)	0.0261 (8)	
H7A	0.1495	0.2136	−0.1630	0.039*	
H7B	0.0376	0.1714	−0.2219	0.039*	
H7C	0.1939	0.1736	−0.2731	0.039*	
C8	0.3222 (4)	0.20639 (12)	0.0623 (4)	0.0214 (7)	
H8A	0.2428	0.2314	0.0514	0.026*	
H8B	0.3961	0.2169	0.0032	0.026*	
C9	0.3872 (4)	0.20533 (12)	0.2131 (4)	0.0212 (7)	
H9A	0.4619	0.2319	0.2281	0.025*	
H9B	0.3117	0.2123	0.2736	0.025*	
C10	0.5278 (4)	0.14069 (13)	0.3512 (4)	0.0197 (7)	
C11	0.5756 (4)	0.17735 (13)	0.4637 (4)	0.0258 (8)	
H11A	0.5662	0.2127	0.4311	0.039*	
H11B	0.6755	0.1705	0.4944	0.039*	
H11C	0.5161	0.1725	0.5382	0.039*	
C12	0.5645 (3)	0.08382 (13)	0.3585 (3)	0.0177 (7)	
C13	0.6640 (4)	0.06293 (14)	0.4569 (4)	0.0231 (8)	
H13	0.7115	0.0843	0.5233	0.028*	
C14	0.6917 (4)	0.00970 (14)	0.4551 (4)	0.0240 (8)	
H14	0.7586	−0.0050	0.5205	0.029*	
C15	0.6207 (3)	−0.02138 (14)	0.3572 (4)	0.0228 (8)	
H15	0.6378	−0.0572	0.3550	0.027*	
C16	0.5222 (3)	0.00235 (13)	0.2610 (3)	0.0192 (7)	
H16	0.4733	−0.0184	0.1940	0.023*	
C17	0.7467 (4)	0.17719 (14)	−0.0524 (4)	0.0258 (8)	
C18	−0.0166 (4)	0.16582 (14)	0.3522 (4)	0.0298 (9)	

### Atomic displacement parameters (Å^2^)

**Table:**

	*U*^11^	*U*^22^	*U*^33^	*U*^12^	*U*^13^	*U*^23^
C1	0.0212 (17)	0.0175 (14)	0.0241 (18)	−0.0001 (12)	0.0006 (13)	0.0005 (12)
C2	0.0260 (18)	0.0214 (16)	0.0240 (19)	0.0052 (13)	0.0029 (15)	0.0050 (13)
C3	0.0282 (19)	0.0299 (18)	0.0200 (18)	0.0050 (14)	−0.0055 (15)	0.0042 (14)
C4	0.0235 (17)	0.0281 (17)	0.0171 (17)	0.0001 (13)	0.0009 (13)	−0.0007 (13)
C5	0.0176 (15)	0.0211 (15)	0.0154 (16)	−0.0013 (12)	0.0071 (12)	−0.0016 (12)
C6	0.0156 (15)	0.0198 (14)	0.0195 (17)	−0.0030 (11)	0.0057 (13)	−0.0021 (12)
C7	0.0258 (18)	0.0248 (16)	0.0249 (19)	−0.0026 (13)	−0.0012 (14)	−0.0059 (14)
C8	0.0268 (17)	0.0133 (14)	0.0228 (18)	−0.0011 (12)	0.0066 (14)	−0.0020 (12)
C9	0.0260 (17)	0.0154 (14)	0.0215 (17)	0.0012 (12)	0.0065 (14)	0.0028 (12)
C10	0.0176 (15)	0.0203 (15)	0.0183 (17)	0.0041 (12)	0.0056 (13)	0.0032 (12)
C11	0.0283 (18)	0.0226 (16)	0.0243 (18)	0.0036 (13)	0.0018 (15)	0.0098 (13)
C12	0.0154 (15)	0.0214 (15)	0.0152 (16)	0.0029 (11)	0.0043 (12)	0.0026 (12)
C13	0.0182 (16)	0.0294 (17)	0.0184 (17)	0.0028 (13)	0.0014 (13)	0.0028 (13)
C14	0.0168 (16)	0.0322 (18)	0.0208 (18)	−0.0048 (13)	−0.0013 (13)	−0.0027 (14)
C15	0.0178 (16)	0.0239 (16)	0.0255 (19)	−0.0032 (12)	0.0050 (14)	−0.0031 (13)
C16	0.0187 (16)	0.0192 (14)	0.0197 (17)	0.0005 (12)	0.0022 (13)	0.0009 (12)
C17	0.0249 (18)	0.0209 (15)	0.0288 (19)	−0.0019 (13)	0.0041 (15)	0.0001 (13)
C18	0.0267 (19)	0.0265 (17)	0.032 (2)	0.0015 (14)	0.0002 (16)	0.0033 (15)
N1	0.0178 (13)	0.0165 (12)	0.0148 (13)	0.0004 (10)	0.0011 (10)	0.0012 (10)
N2	0.0195 (13)	0.0152 (12)	0.0180 (14)	−0.0027 (10)	0.0041 (11)	−0.0002 (10)
N3	0.0171 (13)	0.0151 (12)	0.0186 (14)	0.0015 (10)	0.0032 (11)	0.0033 (10)
N4	0.0165 (13)	0.0174 (12)	0.0150 (13)	0.0008 (9)	0.0025 (10)	0.0004 (10)
O1	0.0240 (13)	0.0339 (13)	0.0252 (14)	−0.0048 (10)	−0.0015 (10)	−0.0036 (10)
O2	0.0260 (13)	0.0203 (11)	0.0262 (13)	−0.0004 (9)	0.0064 (10)	−0.0032 (9)
O3	0.0348 (14)	0.0297 (13)	0.0232 (13)	−0.0033 (10)	0.0076 (11)	0.0051 (10)
O4	0.0273 (13)	0.0284 (12)	0.0222 (13)	0.0030 (10)	0.0085 (10)	0.0059 (10)
O5	0.0302 (14)	0.0403 (14)	0.0255 (14)	−0.0047 (11)	0.0092 (11)	−0.0109 (11)
O6	0.0221 (13)	0.0544 (17)	0.0248 (14)	0.0007 (12)	−0.0011 (11)	0.0122 (12)
Cu1	0.0197 (2)	0.01299 (18)	0.0159 (2)	−0.00029 (14)	−0.00132 (15)	0.00080 (14)
S1	0.0213 (4)	0.0193 (4)	0.0172 (4)	−0.0013 (3)	0.0030 (3)	0.0007 (3)
S2	0.0184 (4)	0.0262 (4)	0.0175 (4)	−0.0022 (3)	0.0029 (3)	−0.0002 (3)
F1	0.0366 (12)	0.0300 (11)	0.0326 (12)	−0.0027 (9)	0.0002 (10)	0.0123 (9)
F2	0.0227 (11)	0.0352 (11)	0.0414 (13)	−0.0044 (8)	−0.0026 (10)	0.0015 (10)
F3	0.0382 (13)	0.0278 (11)	0.0511 (15)	0.0085 (9)	0.0084 (11)	−0.0106 (10)
F4	0.0203 (10)	0.0385 (12)	0.0369 (13)	0.0027 (8)	−0.0053 (9)	−0.0064 (9)
F5	0.0430 (14)	0.0500 (14)	0.0597 (17)	−0.0172 (11)	−0.0004 (13)	0.0275 (13)
F6	0.0374 (13)	0.0294 (11)	0.0646 (17)	0.0069 (10)	−0.0061 (12)	−0.0200 (11)

### Geometric parameters (Å, °)

**Table:**

Cu1—N3	1.941 (3)	C2—C3	1.376 (5)
Cu1—N2	1.949 (3)	C2—H2	0.9300
Cu1—N1	2.011 (3)	C3—C4	1.387 (5)
Cu1—N4	2.016 (3)	C3—H3	0.9300
Cu1—O4	2.474 (3)	C4—C5	1.375 (5)
Cu1—O2	2.564 (3)	C4—H4	0.9300
S1—O3	1.442 (3)	C5—C6	1.494 (5)
S1—O1	1.442 (3)	C6—C7	1.481 (5)
S1—O2	1.453 (3)	C7—H7A	0.9603
S1—C17	1.826 (4)	C7—H7B	0.9603
S2—O6	1.441 (3)	C7—H7C	0.9603
S2—O5	1.442 (3)	C8—C9	1.549 (5)
S2—O4	1.444 (3)	C8—H8A	0.9700
S2—C18	1.828 (4)	C8—H8B	0.9700
F1—C17	1.328 (4)	C9—H9A	0.9700
F2—C17	1.340 (4)	C9—H9B	0.9700
F3—C17	1.339 (4)	C10—C12	1.492 (4)
F4—C18	1.337 (4)	C10—C11	1.486 (5)
F5—C18	1.322 (4)	C11—H11A	0.9608
F6—C18	1.337 (4)	C11—H11B	0.9608
N1—C1	1.342 (4)	C11—H11C	0.9608
N1—C5	1.367 (4)	C12—C13	1.381 (5)
N2—C6	1.287 (4)	C13—C14	1.385 (5)
N2—C8	1.466 (4)	C13—H13	0.9300
N3—C10	1.288 (4)	C14—C15	1.369 (5)
N3—C9	1.465 (4)	C14—H14	0.9300
N4—C16	1.335 (4)	C15—C16	1.392 (5)
N4—C12	1.349 (4)	C15—H15	0.9300
C1—C2	1.383 (5)	C16—H16	0.9300
C1—H1	0.9300		
			
N3—Cu1—N2	83.11 (12)	N2—C6—C5	113.5 (3)
N3—Cu1—N1	164.79 (11)	C7—C6—C5	120.4 (3)
N2—Cu1—N1	81.96 (11)	C6—C7—H7A	109.5
N3—Cu1—N4	81.59 (11)	C6—C7—H7B	109.5
N2—Cu1—N4	164.06 (11)	H7A—C7—H7B	109.4
N1—Cu1—N4	113.52 (12)	C6—C7—H7C	109.5
N3—Cu1—O4	90.25 (10)	H7A—C7—H7C	109.4
N2—Cu1—O4	90.19 (10)	H7B—C7—H7C	109.4
N1—Cu1—O4	86.96 (10)	N2—C8—C9	108.4 (2)
N4—Cu1—O4	94.30 (10)	N2—C8—H8A	110.1
N3—Cu1—O2	90.61 (10)	C9—C8—H8A	110.0
N2—Cu1—O2	88.21 (10)	N2—C8—H8B	110.0
N1—Cu1—O2	91.76 (10)	C9—C8—H8B	110.0
N4—Cu1—O2	87.53 (9)	H8A—C8—H8B	108.4
O4—Cu1—O2	178.07 (8)	N3—C9—C8	107.9 (2)
O3—S1—O1	115.72 (17)	N3—C9—H9A	110.1
O3—S1—O2	114.34 (16)	C8—C9—H9A	110.1
O1—S1—O2	114.89 (15)	N3—C9—H9B	110.2
O3—S1—C17	103.46 (16)	C8—C9—H9B	110.1
O1—S1—C17	102.90 (17)	H9A—C9—H9B	108.4
O2—S1—C17	103.10 (17)	N3—C10—C12	113.1 (3)
O6—S2—O5	115.54 (17)	N3—C10—C11	125.1 (3)
O6—S2—O4	114.61 (15)	C12—C10—C11	121.8 (3)
O5—S2—O4	114.34 (16)	C10—C11—H11A	109.6
O6—S2—C18	103.29 (17)	C10—C11—H11B	109.5
O5—S2—C18	102.91 (16)	H11A—C11—H11B	109.4
O4—S2—C18	103.87 (17)	C10—C11—H11C	109.5
S1—O2—Cu1	138.17 (14)	H11A—C11—H11C	109.4
S2—O4—Cu1	138.10 (15)	H11B—C11—H11C	109.4
C1—N1—C5	118.5 (3)	N4—C12—C13	121.5 (3)
C1—N1—Cu1	130.3 (2)	N4—C12—C10	115.5 (3)
C5—N1—Cu1	111.2 (2)	C13—C12—C10	123.0 (3)
C6—N2—C8	125.5 (3)	C12—C13—C14	118.9 (3)
C6—N2—Cu1	117.1 (2)	C12—C13—H13	120.6
C8—N2—Cu1	115.6 (2)	C14—C13—H13	120.6
C10—N3—C9	124.5 (3)	C15—C14—C13	120.1 (3)
C10—N3—Cu1	117.1 (2)	C15—C14—H14	119.9
C9—N3—Cu1	115.8 (2)	C13—C14—H14	120.0
C16—N4—C12	118.9 (3)	C14—C15—C16	117.9 (3)
C16—N4—Cu1	129.8 (2)	C14—C15—H15	121.1
C12—N4—Cu1	111.3 (2)	C16—C15—H15	121.0
N1—C1—C2	122.4 (3)	N4—C16—C15	122.7 (3)
N1—C1—H1	118.8	N4—C16—H16	118.7
C2—C1—H1	118.8	C15—C16—H16	118.6
C3—C2—C1	118.8 (3)	F1—C17—F2	108.2 (3)
C3—C2—H2	120.7	F1—C17—F3	108.1 (3)
C1—C2—H2	120.6	F2—C17—F3	106.8 (3)
C2—C3—C4	119.5 (3)	F1—C17—S1	112.0 (2)
C2—C3—H3	120.3	F2—C17—S1	111.3 (2)
C4—C3—H3	120.2	F3—C17—S1	110.2 (3)
C5—C4—C3	119.2 (3)	F5—C18—F4	108.1 (3)
C5—C4—H4	120.4	F5—C18—F6	107.9 (3)
C3—C4—H4	120.5	F4—C18—F6	107.1 (3)
N1—C5—C4	121.5 (3)	F5—C18—S2	110.8 (3)
N1—C5—C6	115.3 (3)	F4—C18—S2	111.3 (2)
C4—C5—C6	123.2 (3)	F6—C18—S2	111.5 (3)
N2—C6—C7	126.0 (3)		
			
O3—S1—O2—Cu1	−150.8 (2)	C2—C3—C4—C5	1.7 (6)
O1—S1—O2—Cu1	−13.5 (3)	C1—N1—C5—C4	0.8 (5)
C17—S1—O2—Cu1	97.6 (2)	Cu1—N1—C5—C4	−178.2 (3)
N3—Cu1—O2—S1	−77.9 (2)	C1—N1—C5—C6	179.5 (3)
N2—Cu1—O2—S1	5.2 (2)	Cu1—N1—C5—C6	0.5 (3)
N1—Cu1—O2—S1	87.1 (2)	C3—C4—C5—N1	−1.7 (5)
N4—Cu1—O2—S1	−159.4 (2)	C3—C4—C5—C6	179.7 (3)
O6—S2—O4—Cu1	−5.8 (3)	C8—N2—C6—C7	−1.7 (6)
O5—S2—O4—Cu1	−142.5 (2)	Cu1—N2—C6—C7	−165.8 (3)
C18—S2—O4—Cu1	106.1 (2)	C8—N2—C6—C5	174.8 (3)
N3—Cu1—O4—S2	−10.5 (2)	Cu1—N2—C6—C5	10.7 (4)
N2—Cu1—O4—S2	−93.6 (2)	N1—C5—C6—N2	−7.2 (4)
N1—Cu1—O4—S2	−175.5 (2)	C4—C5—C6—N2	171.5 (3)
N4—Cu1—O4—S2	71.1 (2)	N1—C5—C6—C7	169.5 (3)
N3—Cu1—N1—C1	−164.2 (4)	C4—C5—C6—C7	−11.8 (5)
N2—Cu1—N1—C1	−175.1 (3)	C6—N2—C8—C9	170.5 (3)
N4—Cu1—N1—C1	8.9 (3)	Cu1—N2—C8—C9	−25.1 (3)
O4—Cu1—N1—C1	−84.5 (3)	C10—N3—C9—C8	171.4 (3)
O2—Cu1—N1—C1	97.0 (3)	Cu1—N3—C9—C8	−27.3 (3)
N3—Cu1—N1—C5	14.7 (6)	N2—C8—C9—N3	32.2 (4)
N2—Cu1—N1—C5	3.8 (2)	C9—N3—C10—C12	174.1 (3)
N4—Cu1—N1—C5	−172.2 (2)	Cu1—N3—C10—C12	13.1 (4)
O4—Cu1—N1—C5	94.4 (2)	C9—N3—C10—C11	−4.3 (5)
O2—Cu1—N1—C5	−84.2 (2)	Cu1—N3—C10—C11	−165.4 (3)
N3—Cu1—N2—C6	174.5 (3)	C16—N4—C12—C13	1.0 (5)
N1—Cu1—N2—C6	−8.4 (3)	Cu1—N4—C12—C13	−178.4 (2)
N4—Cu1—N2—C6	158.2 (4)	C16—N4—C12—C10	−179.9 (3)
O4—Cu1—N2—C6	−95.3 (3)	Cu1—N4—C12—C10	0.8 (3)
O2—Cu1—N2—C6	83.6 (3)	N3—C10—C12—N4	−8.9 (4)
N3—Cu1—N2—C8	8.7 (2)	C11—C10—C12—N4	169.6 (3)
N1—Cu1—N2—C8	−174.2 (2)	N3—C10—C12—C13	170.2 (3)
N4—Cu1—N2—C8	−7.6 (6)	C11—C10—C12—C13	−11.2 (5)
O4—Cu1—N2—C8	98.9 (2)	N4—C12—C13—C14	−0.5 (5)
O2—Cu1—N2—C8	−82.1 (2)	C10—C12—C13—C14	−179.6 (3)
N2—Cu1—N3—C10	174.2 (3)	C12—C13—C14—C15	−0.2 (5)
N1—Cu1—N3—C10	163.4 (4)	C13—C14—C15—C16	0.4 (5)
N4—Cu1—N3—C10	−10.2 (3)	C12—N4—C16—C15	−0.8 (5)
O4—Cu1—N3—C10	84.1 (3)	Cu1—N4—C16—C15	178.4 (2)
O2—Cu1—N3—C10	−97.6 (3)	C14—C15—C16—N4	0.1 (5)
N2—Cu1—N3—C9	11.5 (2)	O3—S1—C17—F1	−173.3 (2)
N1—Cu1—N3—C9	0.6 (6)	O1—S1—C17—F1	65.9 (3)
N4—Cu1—N3—C9	−173.0 (2)	O2—S1—C17—F1	−53.9 (3)
O4—Cu1—N3—C9	−78.6 (2)	O3—S1—C17—F2	−52.0 (3)
O2—Cu1—N3—C9	99.6 (2)	O1—S1—C17—F2	−172.8 (3)
N3—Cu1—N4—C16	−174.7 (3)	O2—S1—C17—F2	67.4 (3)
N2—Cu1—N4—C16	−158.3 (4)	O3—S1—C17—F3	66.3 (3)
N1—Cu1—N4—C16	7.1 (3)	O1—S1—C17—F3	−54.5 (3)
O4—Cu1—N4—C16	95.7 (3)	O2—S1—C17—F3	−174.3 (2)
O2—Cu1—N4—C16	−83.7 (3)	O6—S2—C18—F5	−53.1 (3)
N3—Cu1—N4—C12	4.5 (2)	O5—S2—C18—F5	67.5 (3)
N2—Cu1—N4—C12	20.9 (5)	O4—S2—C18—F5	−173.1 (3)
N1—Cu1—N4—C12	−173.6 (2)	O6—S2—C18—F4	−173.4 (3)
O4—Cu1—N4—C12	−85.1 (2)	O5—S2—C18—F4	−52.8 (3)
O2—Cu1—N4—C12	95.5 (2)	O4—S2—C18—F4	66.7 (3)
C5—N1—C1—C2	0.1 (5)	O6—S2—C18—F6	67.1 (3)
Cu1—N1—C1—C2	178.9 (2)	O5—S2—C18—F6	−172.4 (3)
N1—C1—C2—C3	0.0 (5)	O4—S2—C18—F6	−52.9 (3)
C1—C2—C3—C4	−0.9 (5)		
